# Bone Density Changes at the Origin of the Deltoid Muscle following Reverse Shoulder Arthroplasty

**DOI:** 10.3390/jcm13133695

**Published:** 2024-06-25

**Authors:** Antonio Caldaria, Edoardo Giovannetti de Sanctis, Luca Saccone, Angelo Baldari, Danila Azzolina, Luca La Verde, Alessio Palumbo, Francesco Franceschi

**Affiliations:** 1 Department of Orthopaedic and Trauma Surgery, San Pietro Fatebenefratelli Hospital, 00189 Rome, Italy; acaldaria@gmail.com (A.C.); doc.angelobaldari@gmail.com (A.B.); lucalaverde1@gmail.com (L.L.V.); alessio.palumbo@hotmail.it (A.P.); francesco.franceschi@unicamillus.org (F.F.); 2 Faculty of Medicine and Surgery, UniCamillus-Saint Camillus International University of Health and Medical Sciences, 00131 Rome, Italy; 3 Institut Universitaire Locomoteur et du Sport (IULS), Hôpital Pasteur 2, CHU de Nice, 30, Avenue Voie Romaine, 06000 Nice, France; edoardo.giovannettids@gmail.com; 4 Department of Orthopaedics and Traumatology, Fondazione Policlinico Universitario Campus Bio-Medico of Rome, 00128 Rome, Italy; 5 Department of Preventive and Environmental Science, University of Ferrara, 44131 Ferrara, Italy; danila.azzolina@unife.it

**Keywords:** shoulder, arthroplasty, deltoid, bone density, acromion, scapula, clavicle

## Abstract

**Background:** Reverse total shoulder arthroplasty (RSA) significantly impacts deltoid length, tension, and structure. Studies have extensively investigated various modifications in deltoid characteristics, such as perfusion, elasticity, caliber, histological changes, and strength post-RSA. However, to date, there is a notable absence of research evaluating changes in bone mineral density (BMD) at the deltoid muscle origin after the RSA procedure. **Methods:** A retrospective analysis of a consecutive series of RSAs performed between May 2011 and May 2022 was conducted. Inclusion criteria comprised primary RSAs with both preoperative and last follow-up shoulder CT scans and a minimum follow-up of 12 months. Trabecular attenuation measured in Hounsfield units (HU) was calculated using a rapid region-of-interest (ROI) method. BMD analysis involved segmenting three ROIs in both pre- and postoperative CT scans of each patient: the acromion, clavicle, and spine of the scapula. **Results:** A total of 44 RSAs in 43 patients, comprising 29 women and 14 men, were included in this study. The mean follow-up duration was 49 ± 22.64 months. Significant differences were observed between preoperative and postoperative HU values in all analyzed regions. Specifically, BMD increased in the acromion and spine, while it decreased in the clavicle (*p*-values 0.0019, <0.0001, and 0.0088, respectively). **Conclusions:** The modifications in shoulder biomechanics and, consequently, deltoid tension post-implantation result in discernible variations in bone quality within the analyzed regions. This study underscores the importance of thorough preoperative patient planning. By utilizing CT images routinely obtained before reverse shoulder replacement surgery, patients at high risk for fractures of the acromion, clavicle, and scapular spine can be identified.

## 1. Introduction

The Grammont Reverse Shoulder Arthroplasty (RSA) modifies shoulder biomechanics and is based on two principles: the medialization of the Center of Rotation (CoR) to recruit more deltoid fibers and the distalization of the humerus to maintain adequate muscle tension [1].

The preoperative and postoperative conditions of the deltoid muscle are acknowledged as crucial factors influencing surgical outcomes [2,3,4]. There is a variation in the magnitude of the deltoid moment arms between a regular shoulder and an RSA across all three deltoid heads. RSA exhibits a more limited range in the average deltoid moment arms but a higher absolute magnitude throughout the abduction arc when compared to normal shoulders [5]. Various studies have explored changes in deltoid elasticity, muscle fiber caliber, histology, and perfusion following RSA. In elastographic testing, Fisher et al. demonstrated greater stiffness in the deltoid muscle on the operated side compared to the contralateral side [6]. In a separate study, Fisher et al. observed an increased average muscle fiber area (MMFA) six months after RSA [7]. Initially, this muscle hypertrophy led to an enhanced postoperative functional outcome. 

However, this trend appeared to diminish over longer follow-up periods. The underlying reasons for this phenomenon remain debated. Greiner et al. [8] proposed the “theory of deltoid fatigue”, suggesting a time-dependent correlation between progressive degenerative changes in the deltoid muscle and the length of follow-up. Several studies have suggested that the heightened demands on the deltoid muscle following RSA may contribute to a gradual decrease in long-term overhead range of motion (ROM) due to deltoid degenerative changes [9,10,11,12]. In fact, histological studies not only demonstrated the absence of sustained hypertrophy but also revealed a significant decrease in the relative average MMFA by 25.5% one year post-RSA [13]. 

Nevertheless, there is a lack of studies demonstrating whether these changes in the deltoid structure correlate with alterations in bone mineral density (BMD) at its points of origin. 

Studies examining BMD after RSA have primarily focused on the glenoid and proximal humerus, neglecting assessment of the acromion, clavicle, and scapular spine [14,15,16,17]. Bone density assessment typically involves dual X-ray absorptiometry (DXA) scans. Unfortunately, pre-operative DXA scans may not always be available for patients undergoing shoulder arthroplasty. In recent years, the evaluation of Hounsfield units (HU) on computed tomography (CT) scans has shown reliability and accuracy in assessing bone quality, rendering it a viable screening tool for detecting individuals with low BMD [18].

To our knowledge, changes in BMD between preoperative and postoperative RSA have not been assessed. Therefore, our primary objective was to evaluate potential postoperative changes in BMD at the origin points of the deltoid muscle in comparison to the preoperative status. The secondary aim of this study was to determine whether observed BMD changes could be linked to structural alterations in the deltoid muscle, as reported in existing literature. We hypothesized that BMD changes after RSA would correlate with the degree of tension or fatigue in deltoid muscle fibers. Specifically, we anticipated a reduction in BMD associated with degeneration of muscle fibers.

## 2. Materials and Methods

We conducted a retrospective analysis of 508 consecutive RSA performed between May 2011 and May 2022 at the Private Hospital Fatebenefratelli—Villa San Pietro in Rome, Italy. A senior surgeon (F.F.) performed all procedures using a deltopectoral approach and four different implant constructs:

Aequalis Ascend Flex Reverse System 145° (Stryker Corp., Kalamazoo, MI, USA), Equinoxe Reverse 145° (Exactech, Inc., Gainesville, FL, USA), and Comprehensive Shoulder Arthroplasty System (Zimmer Biomet, Warsaw, IN, USA). All systems were implanted following standardized procedures outlined in the manufacturers’ technical manuals. Notably, all utilized implants incorporated a global lateralization of the offset [19].

We extracted demographic information from the electronic medical records, including age at the time of surgery and last follow-up; diagnosis; sex; laterality; and history of previous ipsilateral shoulder surgery.

The inclusion criteria comprised primary RSA for cuff tear arthropathy; massive irreparable rotator cuff tear; osteoarthritis; availability of pre-operative shoulder CT scans; a minimum follow-up of 12 months; and shoulder CT scans at the final follow-up.

The exclusion criteria involved shoulder arthroscanner; revision arthroplasties; RSA for proximal humeral fracture; conversion of anatomic arthroplasty to RSA; instances of dislocation; infection; shoulder CT scans with metal artifacts; avascular necrosis; benign or malignant tumor of the proximal humerus and inflammatory arthropathy. Shoulder CT arthrograms were excluded due to interference from the articular iodinated contrast medium with measurements.

### 2.1. Image Processing

One independent fellowship-trained shoulder surgeon examiner (A.C.) conducted the image processing and analysis using Carestream Vue PACS (picture-archiving communication system) software V12.0 (Carestream Health, Rochester, NY, USA). The analysis employed a rapid region-of-interest (ROI) approach, measuring HU to calculate trabecular attenuation. HU serves as a comparative indicator of radiodensity in CT image interpretation. During CT reconstruction, the absorption/attenuation coefficient of radiation within tissue is utilized to generate a grayscale image [20]. In line with Pickhardt et al.’s research [21], we categorized bone HU values below 100 as indicative of osteoporosis, values between 100 and 160 as indicative of osteopenia, and values above 160 as indicative of normal BMD. The authors employed Kruskal–Wallis tests to compare CT-attenuation values within BMD categories at each vertebral level from T12 to L5.

We segmented three ROIs for BMD analysis: the acromion, clavicle, and spine of the scapula.

**Figure 1:**
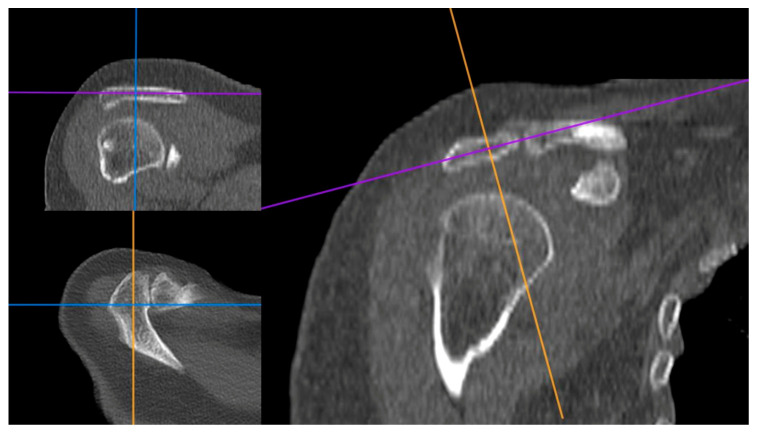
Method for finding the points where the acromion HU value measurement is taken. The center of the acromion bone in axial cut was determined using a coordination facilitated through reformatted coronal and sagittal cut in CT images. Purple line: Axial plane; Blue line: Coronal plane; Orange line: Sagittal plane.

**Figure 2:**
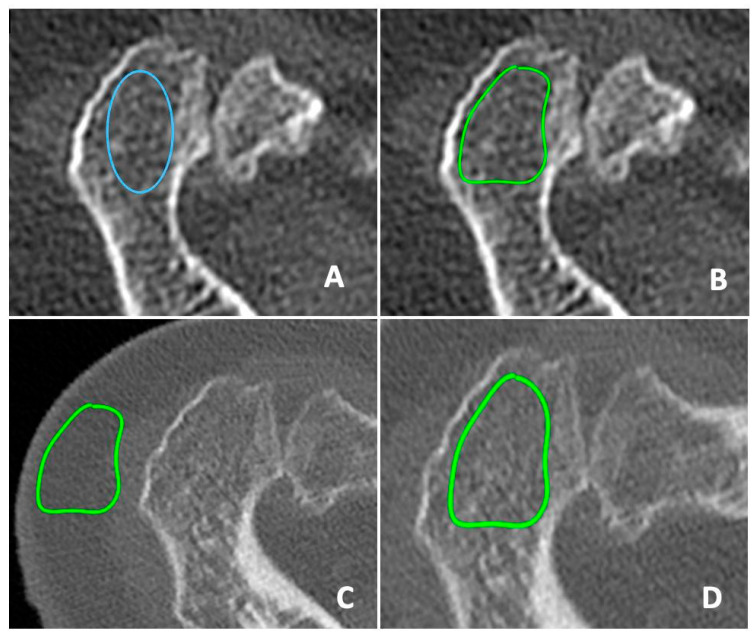
Elliptical approach (blue circle) for ROI measurement (**A**). Segmental approach (green circle) for ROI measurement in preoperative CT scans (**B**). Copy of the ROI identified in the preoperative CT scans in the postoperative CT scans to identify the same section (**C**). ROI measurement in the postoperative CT scans (**D**).

HU measurements were taken by bisecting an axial cut positioned between the acromial process and clavicle, with guidance from reformatted coronal side and sagittal CT images (Figure 1). To measure the HU values of the scapular spine, we utilized the coronal CT cut to avoid cortical bone. Our objective was to consistently measure from the central region of the bone. ROIs were delineated using a segmented approach, providing higher accuracy compared to the elliptical. After identifying the ROI in the preoperative CT images, it was duplicated and superimposed onto the postoperative CT images to ensure measurement consistency (Figure 2).

To manage ROI duplication and superimposition, we performed a three-plane check of the pre- and post-operative CT images using the “3D MPR” option of the PACS software to accurately locate the central portion of the analyzed bone.

We intentionally avoided including cortical bone, focal abnormalities, lesions, and artifacts in our analysis. To isolate trabecular bone for BMD measurements, we utilized adaptive morphological erosion to remove the cortex, focusing solely on trabecular structures. Furthermore, we filtered HU values by retaining all voxels within the range of 0 to 650 HU, while excluding any other HU values in the dataset. The upper threshold of 650 HU was selected as it delineates the interface between cortical and cancellous bone in CT image data. 

### 2.2. Statistical Analysis

Statistical analysis was conducted using GraphPad Prism 10 V10.2.3 (GraphPad Software, Inc., San Diego, CA, USA) The significance threshold was set per convention at alpha = 0.05. Variables were summarized using the mean, standard deviation, and 95% Confidence Intervals (95% CI). The normality distribution was determined using the Shapiro–Wilk and D’Agostino-Pearson tests. For comparisons between groups, the matched-pairs Wilcoxon Signed-Rank test was used for non-normality continuous variables. 

## 3. Results

**Figure 3:**
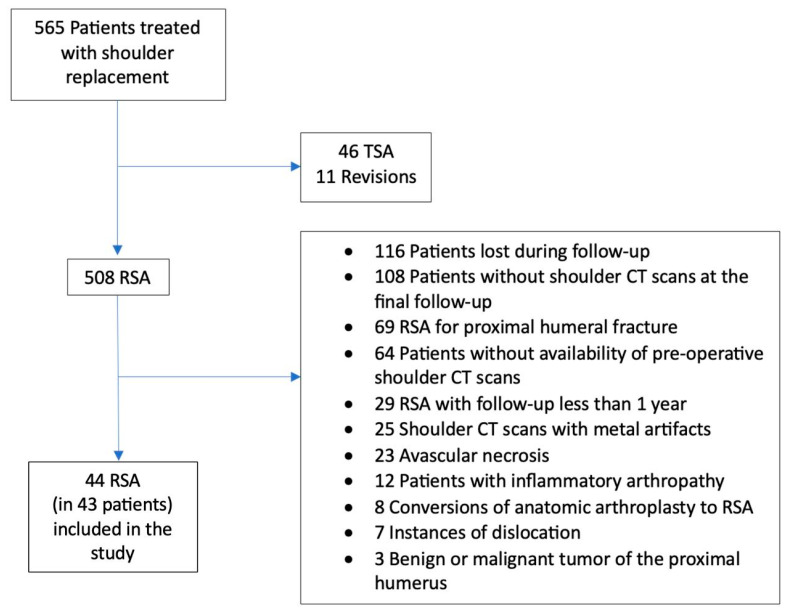
Flowchart of reverse shoulder arthroplasty (RSA) study group.

**Figure 4:**
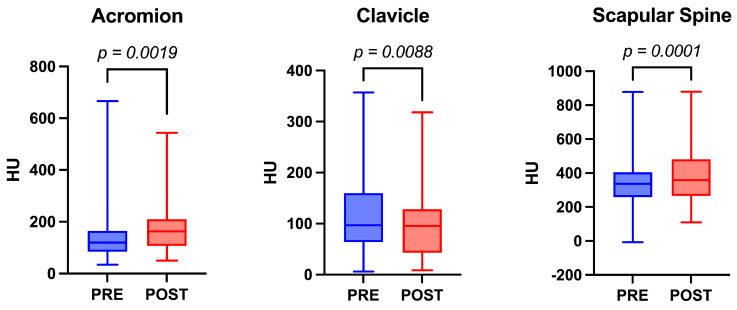
Acromion, Clavicle and Scapular Spine HU values distribution in terms of time (preoperative and postoperative status).

A total of 43 patients and 44 RSA were included in the study (Figure 3). The study group was composed of 29 women (67.5%) and 14 men (32.5%). The mean follow-up was 49 ± 22.64 months. The mean age at the time of surgery was 67.8 ± 8.29 years, and the mean age at the end of the follow-up was 71.9 ± 8.68 years. The preoperative mean HU values for the acromion, clavicle, and scapular spine were 155.9 ± 122.9 HU (range: 118.5–193.3 HU), 117.3 ± 72.34 HU (range: 95.34–139.3 HU), and 347 ± 163.8 HU (range: 297.2–396.8 HU) respectively. At the last postoperative follow-up, the mean HU values for the acromion, clavicle, and spine of the scapula were 179.9 ± 205.5 HU (range: 147.8–212.0 HU), 95.94 ± 62.76 HU (range: 76.86–115.0 HU), and 385.2 ± 161.2 HU (range: 334.9–435.4 HU) respectively. Significant differences were observed between preoperative and postoperative HU values in all analyzed bony regions. Specifically, BMD increased in the acromion and scapular spine, while it decreased in the clavicle (*p*-value 0.0019; <0.0001; and 0.0088, respectively) (Figure 4). The complete descriptive statistics and analysis results are presented in Table 1.

In terms of the acromion, transitions in BMD were observed among the patients: one shifted from osteoporosis to osteopenia; six progressed from osteopenia to normal BMD; and four improved from osteoporosis to normal BMD. Regarding the clavicle, changes in BMD were noted: two patients transitioned from normal BMD to osteopenia; three from normal BMD to osteoporosis; three from osteopenia to osteoporosis; one from osteopenia to normal BMD; and four from osteoporosis to osteopenia. Additionally, two patients experienced scapular fractures postoperatively. Notably, one case exhibited a preoperative scapular spine HU value indicating a normal BMD (190 HU), while the other case showed a severe osteoporosis reading (7 HU).

## 4. Discussion

The condition of the deltoid muscle is recognized as a critical factor influencing surgical outcomes in RSA [2,3,4]. The “theory of deltoid fatigue” [8] posits a time-dependent relationship between the progressive degenerative changes in the deltoid muscle and the length of follow-up. However, studies examining whether these structural changes in the deltoid correlate with alterations in BMD at its origin points are lacking. In this study, we retrospectively evaluated potential postoperative changes in BMD at the deltoid muscle’s origin points in 44 cases of RSA, comparing preoperative and postoperative states. Significant differences were observed between preoperative and postoperative HU values at all origin points of the deltoid muscle.

Muscle degeneration can impact BMD through several pathways. Muscles are crucial for applying mechanical stress to bones, particularly during weight-bearing activities. As muscles deteriorate, their ability to impart this stress onto bones diminishes, resulting in reduced bone density due to the lack of stimulation necessary for bone growth and maintenance [22]. Additionally, there is an intricate interplay between bone and muscle, known as the musculoskeletal unit, wherein hormones and signaling molecules from both tissues mutually influence each other’s health and function [23]. Muscle degeneration can disrupt this interaction, negatively affecting bone density [22]. 

HU has emerged as a valuable tool for evaluating BMD and facilitating the early detection of osteoporosis [18]. Numerous studies have highlighted a strong correlation between BMD, as measured by DXA, and HU values obtained from CT scans [18,21]. These HU values serve as a reliable estimate for regional bone strength and BMD. Pickhardt et al. [21] notably elucidated how bone HU values can be measured and effectively utilized as an alternative to DXA for determining BMD and diagnosing osteoporosis. The authors conducted a study utilizing abdominal CT scans to map the lumbar vertebrae and establish HU values. These values were compared with those obtained from DXA to define HU thresholds for normal bone, osteopenia, and osteoporosis.

Preoperative knowledge of bone quality plays a pivotal role in selecting implants and planning surgeries. It is equally crucial in identifying high-risk patients prone to intraoperative fracture. Notably, in our patient cohort, a preoperative assessment of HU values would have flagged one of the two patients who developed a scapular spine fracture, given their severe osteoporosis. We strongly advocate for a preoperative bone quality assessment across relevant areas to pinpoint patients at a higher risk of fractures. Often, orthopedic surgeons lack comprehensive information about the bone quality of scheduled surgery patients. Conducting CT scans, a routine practice in preoperative shoulder replacements, offers a swift and reliable means to assess osteoporosis in patients.

While most studies concentrate on evaluating preoperative bone quality in the proximal humerus and glenoid [14,15,18,19], these assessments serve distinct purposes: identifying optimal regions for screw placement in the glenoid due to higher bone density [14] and determining suitability for stemless prosthesis in the proximal humerus [24]. Analysis of bone quality by studying CT sequences could also have interesting implications in short-to-medium-term follow-up, considering that some feared complications such as scapular acromial spine fracture would seem to occur in most cases only 9 months after the date of surgery [25]. These observations from the literature would thus seem to indicate that CT studies of bone quality in RSA candidate patients should be performed preoperatively and at postoperative follow-ups within 12 months of the procedure, this being the useful time window for preoperative planning and observations of major postoperative periprosthetic changes.

Surprisingly, no study has explored changes in HU values at the origins of the deltoid muscle. The deltoid muscle originated from the anterior and superior portions of the lateral third of the clavicle (anterior deltoid), from the lateral margin and upper surface of the acromion (lateral deltoid), and from the spine of the scapula (posterior deltoid) [26]. Each part plays a separate role in deltoid function and should be considered distinctly concerning their actions.

The distalizing insertion of the deltoid muscle through RSA induces structural changes. This alteration not only extends the lever arm of the deltoid but also modifies its tension and redistributes force within the muscle [27]. The increased stress imposed on the deltoid muscle after RSA is likely associated with an initial rise in mean MMFA, indicating deltoid muscle hypertrophy. Fisher et al. [6] provided evidence of increased deltoid caliber six months after RSA using contrast-enhanced ultrasound analysis, supporting this hypertrophic response. However, muscle hypertrophy might exhibit temporal limitations and could potentially transition into muscle fatigue owing to overstretching. Greiner et al. [8] established a notable correlation between fatty degeneration of the deltoid muscle and clinical score outcomes. Patients with greater levels of fatty degeneration in the deltoid muscle exhibited lower clinical score results (CS *p*-value = 0.012).

Such shifts in muscle tension or degeneration may influence its attachment to bone, potentially causing alterations in BMD [2]. Our findings align with this notion, as we observed a statistically significant difference in BMD changes at the three investigation points between preoperative and postoperative status. These variations likely correlate with changes in deltoid fiber structure following RSA. 

Greiner et al. [8] documented a progressive degenerative change in the anterior and lateral parts of the deltoid muscle among patients with long-term follow-up after RSA. The same degeneration was not found for the posterior fibers. In addition, this degeneration did not exhibit a correlation with patient age. Notably, their study reported the highest rate of degenerative change in the lateral fibers compared to the anterior fibers. Our study’s findings partially support these observations. We could confirm degeneration in the anterior deltoid muscle, which is often impacted due to the surgical approach. Specifically, we noted a significant decrease in HU values at the clavicle, representing the origin of the anterior deltoid. Contrary to Greiner et al.’s findings, our results did not indicate degeneration in the lateral deltoid, despite its presumed primary stress from lengthening. Surprisingly, we observed a significant increase in HU values in the acromion bone. This divergence in HU values between the acromion and clavicle may stem from three potential hypotheses: first, the degenerative changes in the deltoid muscle might affect only the anterior deltoid muscle; second, initial degeneration may predominantly involve the anterior part, with the lateral part affected subsequently; and third, our utilization of highly lateralized implants likely contributes to increasing tension in the lateral deltoid muscle, potentially preventing its degeneration. 

Several articles have corroborated deltoid degeneration following RSA and confirmed its presence over extended follow-up, resulting in a gradual decline in range of motion (ROM) [9,10,11,28]. This alteration in the muscle resting length is purported to cause deltoid fatigue, leading to a gradual loss of overhead motion approximately 6–8 years post-surgery [9,10,11,28]. Gerber et al. [10] demonstrated a significant reduction in average abduction over time (2.4°/yr), notably declining around nine years post-RSA. However, a recent study did not observe a sudden decline in motion during mid-term follow-up [29]. The outcomes of these studies appear to support our hypothesis regarding the correlation between lateral fiber degeneration and the duration of follow-up. Specifically, the data obtained from our study correspond to a relatively shorter follow-up duration compared to studies in the literature that showcase degeneration in both lateral and anterior deltoid muscle fibers.

### Strengths and Limitations

Several limitations are inherent in this study. It is a retrospective study with a relatively small patient cohort, which may introduce biases typical of such a design. The use of different prosthetic implants, despite being managed by a single skilled surgeon, introduces potential bias into our results due to variations in procedural steps. Moreover, the varying patient follow-up lengths, and the utilization of HU value cut-offs derived from a study primarily focused on the lumbar spine introduce layers of heterogeneity that could influence the results. Additionally, a lack of comparable studies in the literature hinders direct comparisons of the findings.

However, the study also possesses notable strengths compared to existing literature. It stands as the initial investigation to assess changes in bone quality at deltoid origins between preoperative and postoperative stages. The ROIs were evaluated using a segmental method, which, in our estimation, offers superior accuracy compared to the elliptical method. Furthermore, the surgical procedures and measurements were consistently performed by a single skilled surgeon, ensuring an adequate level of uniformity and reliability.

## 5. Conclusions

We present the first evaluation of BMD change at the origin points of deltoid muscle between preoperative and postoperative status after RSA. The alterations in shoulder biomechanics post-RSA have led to discernible differences in bone quality in the examined areas: the acromion, clavicle, and spine of the scapula. Specifically, our observations reveal an enhancement in BMD values of the acromion and scapular spine, coupled with a decrease in BMD values of the clavicle. Findings suggest a potential association between alterations in BMD at these deltoid muscle origin points and variations in muscle tension and/or degeneration. This study underscores the importance of thorough preoperative patient planning. By utilizing CT images routinely obtained before reverse shoulder replacement surgery, patients at high risk of fractures of the acromion, clavicle, and scapular spine can be identified. This identification can be based on preoperative HU values or those that may be observed during follow-up, as informed by the data presented in this article. Further research is essential to provide essential insights into the precise comprehension of the benefits and limitations associated with including routine bone quality assessments utilizing HU in preoperative CT scans to anticipate changes in BMD following RSA at the deltoid muscle origin.

## Figures and Tables

**Table 1:** Descriptive statistics and summarize of comparison of the mean Hounsfield unit values in the preoperative and postoperative status in the analyzed districts. All differences were statistically significant (*).

	ACROMION		CLAVICLE		SCAPULAR SPINE	
	PRE	POST	PRE	POST	PRE	POST
Number of values	44	44	44	44	44	44
Mean	155.9	179.9	117.3	95.94	347	385.2
Std. Deviation	122.9	105.5	72.34	62.76	163.8	161.2
Lower 95% CI of mean	118.5	147.8	95.34	76.86	297.2	334.9
Upper 95% CI of mean	193.3	212	139.3	115	396.8	435.4
Coefficient of variation	78.85%	58.66%	61.65%	65.41%	47.21%	41.84%
Wilcoxon matched pairs signed rank test						
Sum of signed ranks (W)	522		−444		587	
*p* value	0.0019 (*)		0.0088 (*)		0.0001 (*)	

## Data Availability

The dataset is available on request from the authors.
